# Melissa officinalis extract selectively suppresses STAT1 signaling in oral epithelial cells

**DOI:** 10.3389/fimmu.2026.1892750

**Published:** 2026-07-17

**Authors:** Issam Rasheed, Layla Panahipour, Ronald A. Glabonjat, Reinhard Gruber

**Affiliations:** 1 Department of Oral Biology, University Clinic of Dentistry, Medical University of Vienna, Vienna, Austria; 2 Department of Basic Dental Sciences, Faculty of Dentistry, Al-Ahliyya Amman University, Amman, Jordan; 3 Institute of Analytical Chemistry, Faculty of Chemistry, University of Vienna, Vienna, Austria; 4 Austrian Cluster of Tissue Regeneration, Vienna, Austria; 5 Department of Periodontology, School of Dental Medicine, University of Bern, Bern, Switzerland

**Keywords:** anti-inflammatory, caffeic acid, chemokines, cytokines, epithelial cells, *in vitro*, JAK/STAT, melissa officinalis

## Abstract

Oral inflammatory diseases such as oral lichen planus are characterized by dysregulated chemokine production and persistent interferon-associated signaling, with JAK/STAT pathways playing a central role in epithelial-immune crosstalk. Here, we investigated whether Melissa officinalis extract (MOE), a widely used phytomedicine with incompletely defined mechanisms, modulates inflammatory signaling in oral epithelial cells. MOE composition was characterized by HPLC-HRMS, and transcriptomic, RT-qPCR, ELISA, immunofluorescence, and cell-free kinase analyses were employed to define its biological effects in HSC2 and non-transformed epithelial cells. MOE was cytocompatible and selectively attenuated interferon-associated signaling rather than broadly suppressing inflammation. It markedly reduced interferon-stimulated gene expression, including MX1/2, IFIT and OAS family members, STAT1/2, CXCL10, and GBP1, while NF-κB-dependent CXCL8 expression remained unaffected. Mechanistically, MOE reduced JAK2 activity in a cell-free assay and suppressed STAT1 phosphorylation-associated nuclear translocation, supporting modulation of canonical interferon signaling. Consistently, MOE reduced CXCL10 expression at both mRNA and protein levels, and these effects occurred independently of reactive oxygen species modulation. Chemical profiling identified several phenolic acids, among which caffeic acid showed activity in suppressing CXCL10 production. These findings identify MOE as a pathway-selective modulator of interferon-driven inflammatory responses in oral epithelial cells, provide mechanistic insight into its clinical use, and support further investigation of Melissa officinalis-derived preparations as topical strategies for targeted modulation of mucosal inflammation.

## Introduction

Inflammation is a fundamental physiological process that protects against harmful stimuli and promotes tissue repair. However, chronic inflammation involves sustained and dysregulated signalling that drives tissue damage (1). In the oral cavity, such processes underlie diseases including periodontitis and oral mucositis, where amplified inflammatory signalling leads to tissue destruction and pain (2). Key pathways involved include nuclear factor kappa B (NF-κB) and Janus kinase/signal transducer and activator of transcription (JAK/STAT) signalling (3). Moreover, epithelial cells are increasingly recognized as active regulators of mucosal immunity in prototypical inflammatory disorders (4). Developing strategies that selectively modulate these pathways in epithelial cells, rather than broadly suppressing inflammation, remains a major therapeutic challenge in diseases such as oral lichen planus.

Oral lichen planus (OLP) is a chronic immune-mediated disease of the oral mucosa (5). Although its aetiology is not fully understood, JAK/STAT signalling in epithelial cells and leukocytes has emerged as a central contributor to its pathogenesis and a potential therapeutic target (6–8). Current treatment relies on topical corticosteroids, which are effective but primarily symptomatic and associated with adverse effects such as oral candidiasis and mucosal atrophy (9). Notably, targeted inhibition of JAK/STAT signalling, for example with ruxolitinib, has been shown to suppress epithelial inflammation and even reduce disease burden in OLP-like mouse models, highlighting the relevance of this pathway in OLP (10–13). These findings suggest a therapeutic window beyond conventional symptomatic therapy, particularly in the context of phytomedicine.

Oral inflammatory diseases often require supportive home care using topical agents such as mouthwashes. Herbal mouthwash has been shown to improve clinical outcomes in periodontitis and reduce the severity of radiation-induced oral mucositis, supporting its use as an adjunctive topical therapy (2). Plant-derived compounds are increasingly explored as immunomodulatory agents due to their chemical diversity and broad biological activity (14). However, botanical extracts are often broadly classified as anti-inflammatory or antioxidant, although complex phytochemical mixtures can exert selective and context-dependent effects on signalling pathways (15, 16). Notably, various herbs, herbal formulas, natural compounds, and phytochemicals have been reported to regulate biological processes through modulation of the JAK/STAT signalling pathway (17). This concept is particularly relevant for oral lichen planus and related inflammatory disorders, supporting phytomedicine-based topical strategies for pathway-selective modulation of mucosal inflammation.

*Melissa officinalis* (lemon balm) is a widely used medicinal plant with reported anti-inflammatory properties (18). Its chemical composition has been extensively characterized, with aqueous extracts containing phenolic compounds such as caffeic acid, rosmarinic acid, and syringic acid, as well as terpenoids including (-)-caryophyllene oxide (19, 20). However, despite this phytochemical knowledge and the clinical use of *Melissa*-based topical formulations in oral lichenoid conditions (21), the signalling pathways underlying its anti-inflammatory activity in the oral mucosa remain poorly defined. Supporting this relevance, a randomized clinical trial in patients with erosive oral lichenoid reactions showed that *Melissa* gel improved pain, burning sensation, and lesion severity over four weeks (21). Given the central role of interferon-associated JAK/STAT signalling in oral epithelial inflammation, this raises the question of whether *Melissa officinalis* extract (MOE) can selectively modulate this pathway in oral epithelial cells rather than acting solely as general anti-inflammatory agents.

## Materials and methods

### Plant material and extraction method

Melissa officinalis extract (MOE) was prepared using an aqueous maceration method as follows. Dried leaves of Melissa officinalis were purchased from a certified organic herb supplier (Biojoy GmbH, 90402 Nürnberg, Germany). The plant material was ground twice for 30 seconds each using a standard multifunction mill. The ground leaves were then extracted at a concentration of 2% (w/v) in distilled water (2 g per 100 mL). The mixture was agitated at 150 rpm for 10 minutes, followed by a 24-hour incubation period in the dark at room temperature. The extract was subsequently passed through a standard kitchen sieve (pore size approximately 0.5–1 mm) and centrifuged at 3000 × g for 10 minutes. The supernatant was filtered through Whatman filter paper (8 µm pore size), followed by sterile filtration through 0.2 µm syringe filters (Thermo Scientific™ Nalgene™ syringe filters). The final extract was aliquoted and stored at -20 °C. Prior to use, aliquots were thawed at 4 °C.

### Extract characterization

MOE was analysed by reversed-phase HPLC-HRMS (Vanquish Horizon LC and Exploris 120 ESMS from Thermo Scientific) in positive (+3.5 kV) and negative (-2.5 kV) ionization modes over a mass range of m/z 100–750 [resolution was 60,000 full width at half maximum (FWHM)], with data-dependent MS/MS (top four; isolation window 2 m/z; resolution of MS/MS was 15,000 FWHM) fragmentation at a stepped normalized fragmentation energy of 30%. Chromatographic separation of analyte compounds was carried out on an Acclaim C18 column (150 x 2.1 mm, 3 µm particles) under gradient elution conditions using mobile phase A: water including 0.1% (v/v) formic acid and B: acetonitrile/water (9 + 1, v/v) including 0.1% (v/v) formic acid with the following gradient: 0–3 min 5% B; 3–15 min 5-95% B; 15–20 min 95% B; 20-20.1 min 95-5% B; 20.1–25 min 5% B. Column temperature was 30 °C and injection volume was 5 µL. Water blanks were interspersed for background subtraction. Base peak chromatograms (BPC) from ESI+ and ESI- modes were overlaid to visualize the overall chemical profile of the extract. The most abundant peaks in each ionization mode were annotated by matching retention time, accurate mass (± 3 ppm) and fragmentation spectra to the identified compound list. Compound identification was achieved by matching MS1 and MS2 spectra against ChemSpider, mzCloud, and in-house databases. Features with signal-to-blank ratios >=5 were retained. A compound identification table was compiled listing the top 10 most abundant compounds in each ionization mode based on integrated peak area, including name, molecular formula, retention time, measured m/z, mass error (ppm), and confidence level. To assess extraction reproducibility, all compounds with Level 2 identification confidence (full database matches) in both positive and negative ionization modes were subjected to statistical analysis. For each compound, mean signal intensities were calculated from duplicate injections of three independent extract batches. One-way repeated measures ANOVA was performed separately for ESI+ and ESI- datasets to evaluate batch-to-batch variability, with statistical significance set at p < 0.05.

### Cell culture

The oral squamous cell carcinoma cell line HSC2 was obtained from the Health Science Research Resources Bank (Sennan, Japan). Cells were cultured in high-glucose Dulbecco’s Modified Eagle Medium (DMEM; Sigma Aldrich, St. Louis, MO, USA) supplemented with 10% fetal calf serum (FCS; Bio&Sell GmbH, *Nürnberg*, Germany) and antibiotics (Invitrogen Corporation, Carlsbad, CA, USA). Primary oral epithelial cells were isolated from the gingival epithelial layer obtained from extracted third molars of one donor who provided written informed consent (EK NR 631/2007). The isolated cells were cultured in keratinocyte growth medium (PromoCell, Heidelberg, Germany). Cells were maintained under standard culture conditions at 37 °C in a humidified atmosphere containing 5% CO_2_.

### Cell viability

Cells were seeded in culture plates and allowed to adhere overnight. The next day, cells were exposed to different concentrations of MOE for 24 h. Cell viability was assessed using the MTT assay. After treatment, MTT solution was added for 2 h at 37 °C, and the resulting formazan crystals were dissolved in DMSO. Absorbance was measured at 570 nm using a BioTek Synergy HTX multimode reader (Agilent, Santa Clara, CA, USA). Viability was expressed relative to untreated control cells, set as 100%. In parallel, cells treated with 5% (v/v) MOE for 24 h were stained using a live/dead cell staining kit (Enzo Life Sciences, Lausen, Switzerland). Fluorescence images were acquired using an Echo Revolve R4 microscope, and live/dead cells were quantified from at least three independent fields per condition. Cell viability was additionally assessed by Trypan blue exclusion. Briefly, following treatment, cells were mixed with 0.4% Trypan blue solution (Sigma-Aldrich, St. Louis, MO, USA) at a 1:1 ratio. After incubation for 2 minutes at room temperature, microscopic images were taken.

### RNA sequencing and differential gene expression analysis

Total RNA was isolated from HSC2 epithelial cells using the GeneMATRIX Universal RNA Purification Kit with on-column DNase digestion (EURX, Gdańsk, Poland). RNA quality and concentration were assessed using a Bioanalyzer 2100 (Agilent) and Qubit fluorometer (Invitrogen), respectively. Sequencing libraries were prepared from total RNA at the Core Facility Genomics, Medical University of Vienna, using the QuantSeq 3’ FWD protocol version 2 with unique dual indices (Lexogen). The number of PCR cycles for library amplification (15 cycles) was determined by qPCR according to the manufacturer’s instructions. Libraries were quality-controlled on a Bioanalyzer 2100 (Agilent) using a High Sensitivity DNA Kit to confirm correct insert size and quantified using the Qubit dsDNA HS Assay (Invitrogen). Pooled libraries were sequenced on a P3 flow cell using a NextSeq 2000 instrument (Illumina) in 1 × 75 bp single-end sequencing mode, generating an average of 12 million reads per sample. Raw sequencing data in FASTQ format were generated using the Illumina bcl2fastq command line tool (v2.19.1.403) and the Lexogen idemux tool for optimal demultiplexing of long unique dual indices. Reads were trimmed and filtered using cutadapt (v2.8) to remove polyA tails, discard reads containing ambiguous bases (N’s), and trim bases with a quality score below 30 from the 3’ ends. After trimming, an average of 8.7 million reads per sample remained. Trimmed reads were aligned to the human reference genome (GRCh38) with Gencode 29 annotations using STAR aligner (v2.6.1a) in two-pass mode. Raw read counts per gene were quantified by STAR. Differential gene expression analysis was performed using DESeq2 (v1.40.2) with the lfcShrink method for log2 fold change estimation. Genes with an adjusted p-value (false discovery rate) < 0.05 were considered differentially expressed. The raw RNA sequencing data generated during this study have been deposited in the European Nucleotide Archive (ENA) under BioProject accession PRJEB114350 (study accession ERP194657).

### Volcano plot and gene set enrichment analysis

Volcano plots were generated using the online tool VolcaNoseR. Genes were classified as up- or downregulated based on a minimum log_2_ fold change of 1.5 and a −log_10_ p-value threshold of 2.0, and these gene sets were selected for subsequent analyses. Heatmaps were created in R (www.R-project.org; accessed on 20 September 2024) using genes that met the significance criterion of an adjusted p-value < 0.05. Functional enrichment analysis was conducted with STRING, which integrates multiple databases. All sequencing-based datasets are provided in the **Supplementary Files**.

### Reverse transcription quantitative real-time PCR and Immunoassay

HSC2 cells were exposed to 5% (v/v) MOE for six hours in the presence of IL-1β and TNF-α. Untreated cells served as a negative control, while cells treated with IL-1β and TNF-α alone served as a positive control. For IFNγ-stimulation experiments, cells were treated with IFNγ in the presence or absence of 5% (v/v) MOE for six hours. Total RNA was extracted using the GeneMATRIX Universal RNA Purification Kit (EURx Ltd, Gdańsk, Poland). Complementary DNA (cDNA) was synthesized by reverse transcription of total RNA using the LabQ Reverse Transcription Kit (Labconsulting, Vienna, Austria). Quantitative PCR was performed using the LabQ qPCR Kit (Labconsulting, Vienna, Austria) on a CFX Connect Real-Time PCR Detection System (Bio-Rad Laboratories, Hercules, CA, USA). The primer sequences used were as follows: hCXCL10 (F): TGCCATTCTGATTTGCTGCC, hCXCL10 (R): TGCAGGTACAGCGTACAGTT, hCXCL8 (F): AACTTCTCCACAACCCTCTG, hCXCL8 (R): TTGGCAGCCTTCCTGATTTC, hGBP1 (F): AGGAGTTCCTTCAAAGATGTGGA, hGBP1 (R): GCAACTGGACCCTGTCGTT, and h18S (F): CCGATTGGATGGTTTAGTGAG, h18S (R): AGTTCGACCGTCTTCTCAGC. The amount of each specific mRNA was normalized to the housekeeping gene 18S using the ΔΔCt method. RT-qPCR data are presented relative to the unstimulated control, which was set to 1.0 in all analyses. To confirm inhibition of CXCL10 gene expression, CXCL10 protein levels in cell culture supernatants were measured by enzyme-linked immunosorbent assay (ELISA) using a DuoSet kit (R&D Systems, Minneapolis, MN, USA) according to the manufacturer’s instructions.

### Immunofluorescence staining

HSC2 cells and primary oral epithelial cells were grown on Millicell EZ slides (Merck KGaA, Darmstadt, Germany). Prior to treatment, cells were serum-starved overnight. Cells were then treated for 1 hour with 5% (v/v) MOE in the presence or absence of IFNγ, or with IL-1β and TNF-α where indicated. Untreated cells served as a negative control, and cytokine-stimulated cells without MOE served as positive controls. Following treatment, cells were washed with phosphate-buffered saline (PBS) and fixed with 4% paraformaldehyde for 15 minutes at room temperature. Additional fixation using ice-cold absolute methanol was conducted as per company protocol. Cells were then blocked with 1% bovine serum albumin (BSA; Sigma Aldrich, St. Louis, MO, USA) for 60 minutes at room temperature. The primary antibody against phosphorylated STAT1 (pSTAT1, Tyr701, 58D6, Rabbit mAb, 1:50; Cell Signaling Technology, CST, Cambridge, UK, #9167) was added and incubated overnight at 4 °C. After washing, cells were incubated with the secondary antibody, goat anti-rabbit IgG conjugated to Alexa Fluor 488 (1:1000; Cell Signaling Technology, CST, Cambridge, UK, #4412), for 60 minutes at room temperature in the dark. Images were captured using an Echo Revolve R4 fluorescence microscope (San Diego, CA, USA).

### Compounds and JAK2 activity assay

Compounds that were identified as Melissa officinalis constituents were obtained as follows: chlorogenic acid (Sigma-Aldrich, St. Louis, MO, USA; Catalog #C3878), syringic acid (TargetMol, Boston, MA, USA; Catalog #T2883), caryophyllene oxide (TargetMol, Boston, MA, USA; Catalog #T2724), and caffeic acid (Sigma-Aldrich; Catalog #C8221). Candidate compounds were tested for effects on CXCL10 secretion after inflammatory stimulation, and cytocompatibility was assessed at the tested concentrations by MTT assay. Inhibition of JAK2 kinase activity by MOE was assessed using the JAK2 (Janus Kinase 2) Assay Kit (BPS Bioscience, San Diego, CA, USA; Catalog #79520), following the manufacturer’s protocol. Activity was normalized to the enzyme control and no-enzyme control.

### Reactive oxygen species detection

Intracellular ROS levels were assessed using the CellROX Green Reagent (Invitrogen, Catalog No. C10444) according to the manufacturer’s protocol. HSC2 cells were grown on Millicell EZ slides. Following treatment, cells were incubated with 5 µM CellROX Green Reagent for 30 minutes at 37 °C in the dark. Cells were then washed with PBS, fixed with 4% paraformaldehyde. Fluorescence images were captured using an Echo Revolve R4 fluorescence microscope (San Diego, CA, USA). Untreated cells served as a negative control for basal ROS levels. Cells treated with 5% MOE, 10 µM antimycin A as a positive control for ROS induction, and 1 mM N-acetylcysteine (NAC) as a negative control for ROS scavenging.

### Statistical analysis

Data were obtained from independent experiments and analyzed according to the experimental design and data characteristics. Normality was assessed using the Shapiro–Wilk test. Parametric analyses were applied to normally distributed datasets, whereas non-parametric tests were used for datasets that did not meet the normality assumption. For cytotoxicity (MTT assay) and batch-to-batch consistency analyses, comparisons among multiple groups were performed using one-way ANOVA. For RT-qPCR and ELISA datasets involving two-group comparisons, the Mann–Whitney U test was used. For NAC rescue experiments involving multiple treatment groups, gene expression data were analyzed using the Kruskal–Wallis test. Statistical analyses were performed using GraphPad Prism v9 (La Jolla, CA, USA), and statistical significance was defined as *p* < 0.05.

## Results

### Extract characterization

Three independently prepared MOE batches were analysed by HPLC-HRMS to assess extraction reproducibility. Batch-to-batch consistency was evaluated across identified compounds in both ESI+ and ESI- modes (**Supplementary Figure 1**). Mean peak areas did not differ significantly between batches in either ESI^+^ (F = 2.434, p = 0.091) or ESI^-^ (F = 2.238, p = 0.085), indicating consistent chemical profiles across preparations. Batch identity accounted for less than 4% of total variance in both ionization modes (ESI^+^: R² = 0.027; ESI^-^: R² = 0.032). Full batch consistency data are provided in **Supplementary Table 1**. The phytochemical profile of MOE was determined in positive and negative ionization modes. Base peak chromatograms revealed several major annotated compounds, including corchorifatty acid F, syringic acid, caffeic acid, and oxophytodienoic acid. Additional abundant constituents included tryptophan, hexopyranosyloxy-oxocyclopentyl acetic acid, malic acid, caryophyllene oxide, 10-HDA, and 2-furoylglycine (**Table 1**). Ferulic acid and further annotated peaks are listed in **Supplementary File 1**. Among the annotated constituents, caffeic acid, syringic acid, and caryophyllene oxide are of particular interest, as related phenolic acids and terpenoids have been reported to interfere with JAK/STAT signaling.

**Table 1:** Major compounds identified in MOE and their known bioactivity.

Compound	Formula	Database match	Class	Known bioactivity
Caffeic acid	C9H8O4	>99%	Phenolic acid	Potential JAK/STAT inhibitor (22, 23)
Caryophyllene oxide	C15H24O	93%	Terpenoid/sesquiterpene	Potential JAK/STAT inhibitor (24, 25)
Syringic acid	C9H10O5	89%	Phenolic acid	Potential JAK/STAT inhibitor (26, 27)
Corchorifatty acid F	C18H32O5	99%	Oxylipin	Antioxidant effects; modulates inflammatory pathways (28, 29)
Oxophytodienoic acid (OPDA)	C18H28O3	96%	Oxylipin/jasmonate	Anti-inflammatory and antioxidant effects in immune and immune resident cell models (30, 31)
Tryptophan	C11H12N2O2	>99%	Amino acid	Precursor to serotonin and kynurenine pathway; immunomodulatory
Hexopyranosyloxy-oxocyclopentyl acetic acid	C18H28O9	94%	Glycosylated jasmonate	Anti-inflammatory effect in gastrointestinal intestinal models via NF-κB (32, 33)
Malic acid	C4H6O5	95%	Organic acid	Energy metabolism; mild antioxidant
10-HAD	C10H18O3		Fatty acid (hydroxy-decenoic acid)	Royal jelly component; anti-inflammatory; modulates cytokine production
2-Furoylglycine	C7H7NO4	68%	Glycine conjugate	Microbial or mammalian metabolite; unclear bioactivity

### Candidate bioactive compounds in MOE

HPLC-HRMS analysis identified several major constituents of the aqueous MOE (**Table 1**). The main elution window contained diverse compound classes, including phenolic acids, terpenoids, oxylipins, amino acid derivatives, and fatty acid-related metabolites. Several constituents of MOE have been reported to interfere with JAK/STAT-associated signaling, providing a plausible but not definitive molecular basis for the observed effects. Caffeic acid, a phenolic acid, has been reported to modulate JAK/STAT-associated signaling and reduce CXCL10 expression (22, 23). Similarly, (−)-caryophyllene oxide, a sesquiterpene, has been associated with reduced STAT1/3 signaling and anti-inflammatory effects involving JAK/STAT-dependent pathways (24, 25). Syringic acid also displays anti-inflammatory activity and has been reported to modulate STAT3 signaling and chemokine expression (26, 27). Collectively, these compounds may contribute to the attenuation of interferon-driven gene expression observed in MOE-treated cells; however, their individual contributions within the crude extract remain to be defined.

**Figure 1:**
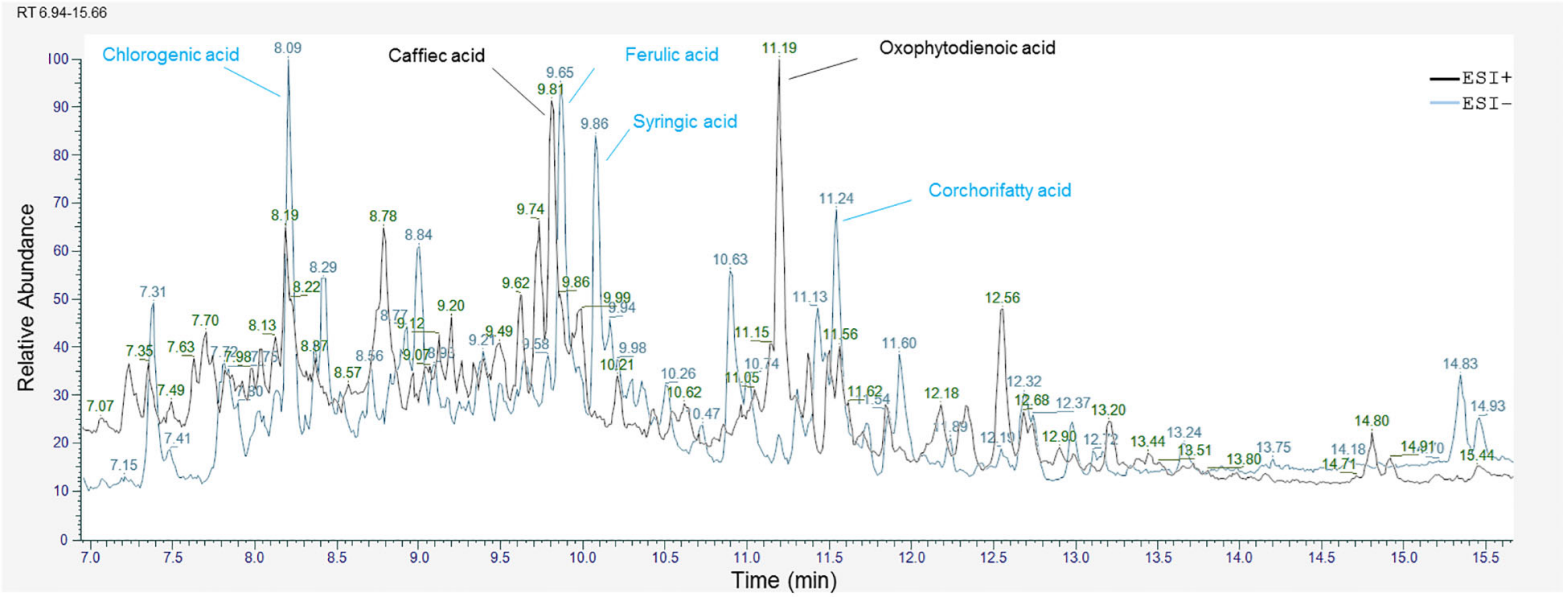
Base peak chromatograms (BPC) of MOE in positive and negative ionization modes. Representative HPLC-HRMS base peak chromatograms of a single extract batch analyzed in positive ion mode (black trace) and negative ion mode (blue trace). Chromatograms are overlaid to visualize the overall chemical profile of the extract. Peaks in the base peak chromatogram were annotated based on RT alignment, accurate masses, isotopic pattern match, and fragmentation pattern comparison with databases, followed by area ranking, peak rating, and chemical plausibility of the formula.

The base peak chromatogram (BPC) displays the most intense ion at each retention time regardless of compound identity, while the compound table ranks compounds by integrated peak area across the entire run. Therefore, a compound with a very sharp, well-resolved peak may appear prominent in the BPC but have a lower total area compared to a compound with a broader peak or lower ionization efficiency. Conversely, a compound with high total area may elute as a broad peak or co-elute with other compounds, making it less visually prominent in the BPC. These differences reflect the complementary nature of the two representations rather than any inconsistency in the data (**Figure 1**).

### MOE does not affect the viability of HSC2 cells

**Figure 2:**
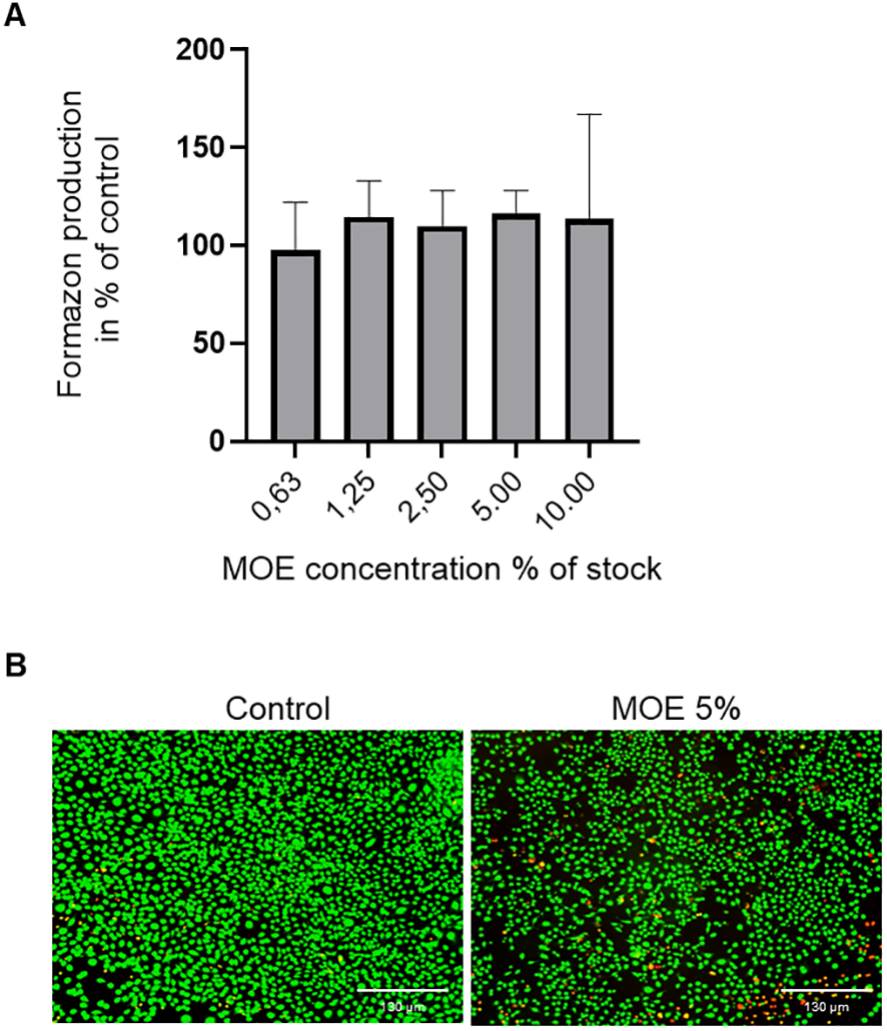
Cytocompatibility of MOE in HSC2 cells. HSC2 cells were exposed to increasing concentrations of MOE for 24 h. **(A)** Cell viability was assessed by MTT assay and expressed as a percentage relative to the untreated control. Data are presented as median ± SD (n = 4 independent experiments). **(B)** Representative live/dead staining images of HSC2 cells treated with 5% (v/v) MOE (green: live cells; red: dead cells, scale bar = 130 µm). The 5% (v/v) MOE concentration was selected for subsequent experiments based on its cytocompatibility and biological activity.

To exclude potential cytotoxic effects at the concentration used in subsequent experiments, HSC2 cells were exposed to five different concentrations of MOE for 24 hours. No significant effects on cell viability were observed within the tested concentration range, as assessed by MTT formazan formation (**Figure 2A**). These findings were confirmed by live/dead staining (**Figure 2B**) and Trypan blue staining (**Supplementary Figure 2**). Collectively, these data demonstrate that treatment with 5% (v/v) MOE from the stock solution (2 g leaves/100 mL) does not significantly affect HSC2 cell viability, supporting its use for subsequent mechanistic studies.

### Transcriptomic effects of MOE

**Figure 3:**
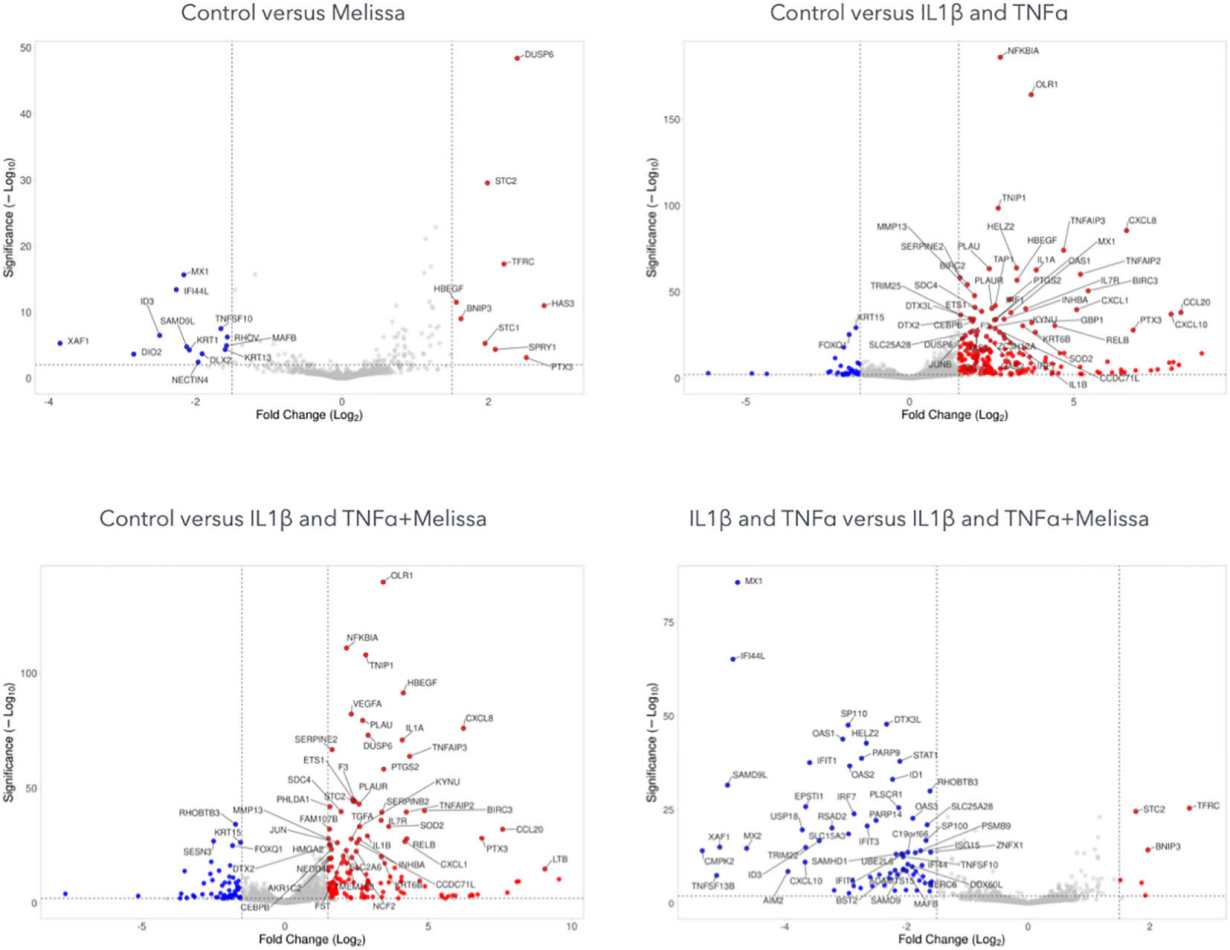
Transcriptomic effects of MOE under basal and inflammatory conditions in HSC2 cells. Volcano plots showing differential gene expression. While MOE alone had only modest transcriptomic effects, mainly marked by increased STC1/2 and DUSP6, together with reduced MX1 and KRT1/13 expression, IL-1β and TNF-α induced the expected prominent inflammation-associated gene signature. Notably, MOE greatly decreased the canonical interferon-signaling genes including MX1/2, IFIT1/3/5, OAS1–3, ISG15, RSAD2, and IFI44/IFI44L, together with regulatory factors such as STAT1, STAT2, and IRF7, indicating that MOE attenuates the inflammatory interferon/JAK-STAT response rather than activating it. This suppressive effect also included chemokines such as CXCL10 and CXCL11, and immune modulators including TNFSF10 and LGALS9.

To characterize the transcriptional response to MOE, HSC2 cells were treated with 5% (v/v) MOE alone, inflammatory cytokines (IL-1β and TNF-α), or their combination for six hours. MOE alone induced modest but selective transcriptional changes, with 13 genes downregulated and 9 upregulated. Downregulated genes included interferon-associated markers (MX1, IFI44L, TNFSF10, XAF1, SAMD9L), indicating suppression of an interferon-related signature, along with epithelial differentiation markers (KRT1, KRT13, DLX2, NECTIN4). Upregulated genes (DUSP6, SPRY1, HBEGF, STC1/2, BNIP3, HAS3, PTX3, TFRC) were associated with growth factor signaling, stress adaptation, and cytoprotective responses, suggesting a distinct regulatory program (**Figure 3**).

### Inflammatory stimulation induces a broad transcriptional response

Stimulation with IL-1β and TNF-α triggered a robust inflammatory response characterized by activation of NF-κB–associated genes (NFKBIA, TNFAIP3, TNIP1/3, BIRC2/3, RELB, IL1A/B, TNF, PTGS2, CEBPB) and strong induction of chemokines (CXCL1–3, CXCL8, CXCL10/11, CCL20, CCL5, CSF3, SAA1/2). In parallel, a pronounced interferon-stimulated gene (ISG) signature was observed, including MX1/2, OAS1–3, IFIT1/3/5, IFI44L, ISG15, RSAD2, USP18, STAT1, IRF1/7, and GBP family members, indicating activation of a secondary interferon-associated program. Genes linked to matrix remodeling and repair (MMP9/13, PLAU/R, HAS3, ICAM1, ITGA5, PDGFB, TGFA, HBEGF) were also upregulated (**Figure 3**).

### MOE selectively suppresses interferon-associated signaling

Co-treatment with MOE markedly and selectively counteracted the inflammatory transcriptional program induced by IL-1β/TNF-α, representing a central finding of this study. The most prominent effect was the broad downregulation of canonical interferon-responsive genes, including MX1/2, IFI44/IFI44L, IFIT1/3/5, OAS1–3, ISG15, RSAD2, USP18, STAT1/2, IRF7, PARP9/12/14, TRIM22, UBE2L6, and SAMD9/SAMD9L. This response was accompanied by reduced expression of inflammatory and immune effector genes such as CXCL10, CXCL11, TNFSF10, TNFSF13B, AIM2, and GBP1/4, indicating that MOE suppresses not only individual cytokine-responsive genes but a coordinated interferon/JAK-STAT-associated inflammatory network. In contrast, only a limited subset of genes, including STC1/2, BNIP3, TFRC, and TNFRSF10D, was upregulated upon MOE co-treatment. Together, these data indicate that MOE does not broadly alter the transcriptome but rather selectively attenuates the IL-1β/TNF-α-driven inflammatory state in HSC2 cells, with prominent suppression of the interferon/STAT1–STAT2 axis (**Figure 3**).

### MOE selectively dampens an interferon-associated transcriptional program

**Figure 4:**
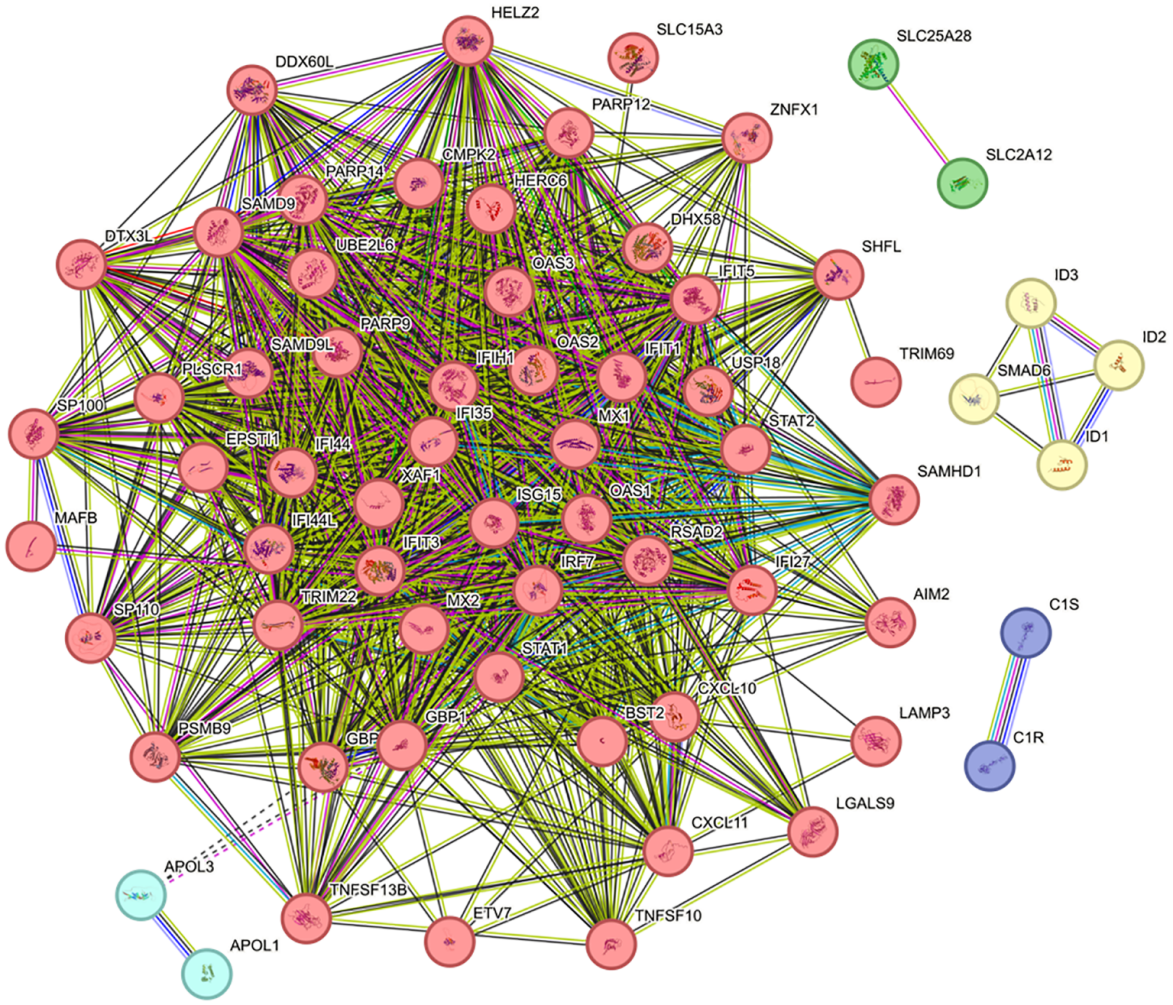
MOE selectively dampens an interferon-associated transcriptional program. STRING analysis of differentially expressed genes in IL-1β/TNF-α-stimulated HSC2 cells treated with or without MOE revealed a tightly connected cluster enriched for interferon-associated genes. Additional regulated genes were related to epithelial differentiation, transcriptional control, apoptosis, and cell adhesion/signaling. Line thickness indicates interaction confidence. These data support preferential suppression of an interferon-associated transcriptional program by MOE.

STRING network analysis further illustrated that the genes downregulated by MOE in IL-1β/TNF-α-stimulated HSC2 cells form a tightly interconnected transcriptional network enriched for interferon-associated pathways. This cluster included canonical interferon-stimulated genes such as CXCL10/11, OAS1/2/3, IFIT1/3/5, IFI44L, MX1/2, SAMD9L, XAF1, TRIM6, and TNFSF10, supporting selective suppression of an interferon-driven program by MOE. In addition, genes related to epithelial differentiation and keratinocyte biology, including KRT1, KRT13, KRT16, and SCGB1A1, were present, alongside transcriptional regulators such as GATA3, CEBPA, E2F2, ID2, ID3, and PBX1. Smaller gene groups associated with apoptosis (BMF, HRK) and cell adhesion or signaling (VAV3, EFNB3, ADAMTS5, ADAM23, NECTIN4) were also identified. In addition, MOE reduced smaller clusters including ID1/2/3/Smad6, C1S/C1R and APOL1/3. Together, these findings indicate that MOE primarily dampens an interferon-associated transcriptional program while also modulating pathways related to epithelial biology and cellular signaling (**Figure 4**).

### Selective inhibition of CXCL10 expression by Melissa officinalis in HSC2 cells

**Figure 5:**
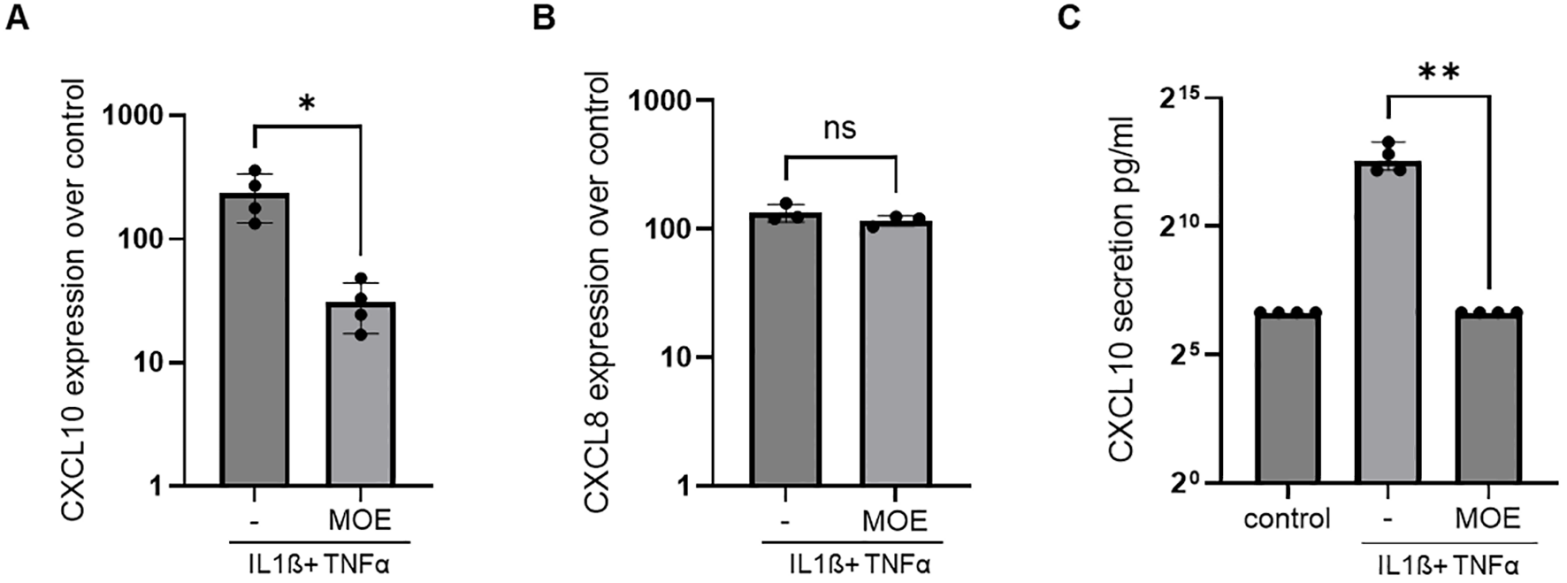
Selective inhibition of CXCL10 expression by MOE. HSC2 cells were stimulated for six hours with IL-1β and TNF-α in the presence or absence of 5% (v/v) MOE. **(A)** CXCL10 and **(B)** CXCL8 mRNA expression measured by RT-qPCR. **(C)** CXCL10 protein levels in cell culture supernatants measured by ELISA following 5% (v/v) MOE treatment for six hours. Relative mRNA expression was calculated as fold change normalized to the unstimulated control (set to 1.0). CXCL10 secretion values are presented on a logarithmic scale due to low CXCL10 levels in control samples. Data are presented as median ± SD (**A, C**, *n* = 4; **B**, *n* = 3 independent experiments). (*p < 0.05, **p < 0.01, ns, not significant.).

To validate the RNA sequencing findings, CXCL10 and CXCL8 expression were assessed by RT-qPCR in IL-1β/TNF-α-stimulated HSC2 cells treated with 5% (v/v) MOE. MOE significantly reduced *CXCL10* mRNA expression, whereas *CXCL8* remained unaffected (**Figures 5A, B**). Consistently, CXCL10 protein secretion was also significantly decreased following MOE treatment (**Figure 5C**). These findings confirm that MOE selectively suppresses CXCL10 at both mRNA and protein levels without broadly inhibiting CXCL8 expression.

### MOE suppresses STAT1 nuclear translocation and CXCL10 expression

**Figure 6:**
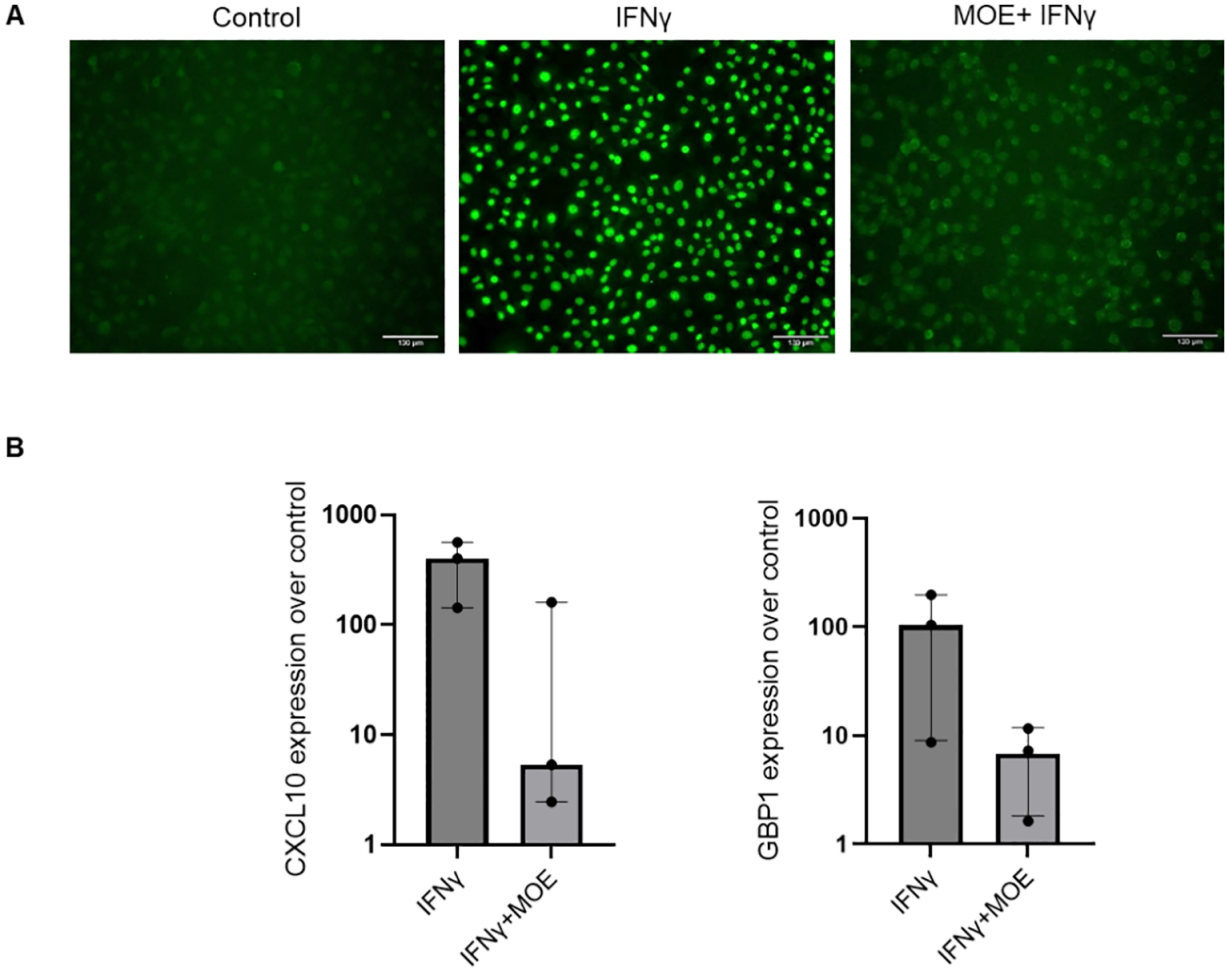
MOE suppresses STAT1 nuclear translocation and interferon-associated gene expression. **(A)** Representative immunofluorescence images of pSTAT1 staining (green) in HSC2 cells stimulated with IFNγ with or without 5% (v/v) MOE for 1 h. MOE reduced the cytokine-induced nuclear pSTAT1 signal. Scale bar = 130 µm. **(B)** CXCL10 and GBP1 mRNA expression measured by RT-qPCR in HSC2 cells stimulated with IFNγ with or without 5% (v/v) MOE for 6 h. Data are presented as median ± SD (n = 3 independent experiments).

To further explore the underlying mechanism, phosphorylated STAT1 nuclear translocation was examined by immunofluorescence. In untreated cells, pSTAT1 was barely detectable, whereas IFNγ stimulation induced pronounced STAT1 phosphorylation and nuclear localization. Co-treatment with MOE markedly reduced both STAT1 phosphorylation-associated signal and nuclear localization (**Figure 6A**). To confirm the functional relevance of this effect, CXCL10 and GBP1 expression were assessed after IFNγ stimulation. IFNγ strongly induced both interferon-responsive genes, and this response was reduced by MOE co-treatment (**Figure 6B**). These findings support inhibition of STAT1 signalling as a key mechanism underlying the selective suppression of interferon-responsive genes by MOE. To confirm that the observed effects were not restricted to the transformed HSC2 model, STAT1 activation was further evaluated in primary oral keratinocytes under the same experimental conditions. Immunofluorescence analysis demonstrated that MOE similarly reduced pSTAT1 nuclear translocation in primary cells, consistent with the findings observed in HSC2 cells (**Supplementary Figure 3**). These results indicate that MOE-mediated modulation of STAT1 signalling is reproducible in non-transformed oral epithelial cells.

### MOE inhibits JAK2 kinase activity in a cell-free assay

**Figure 7:**
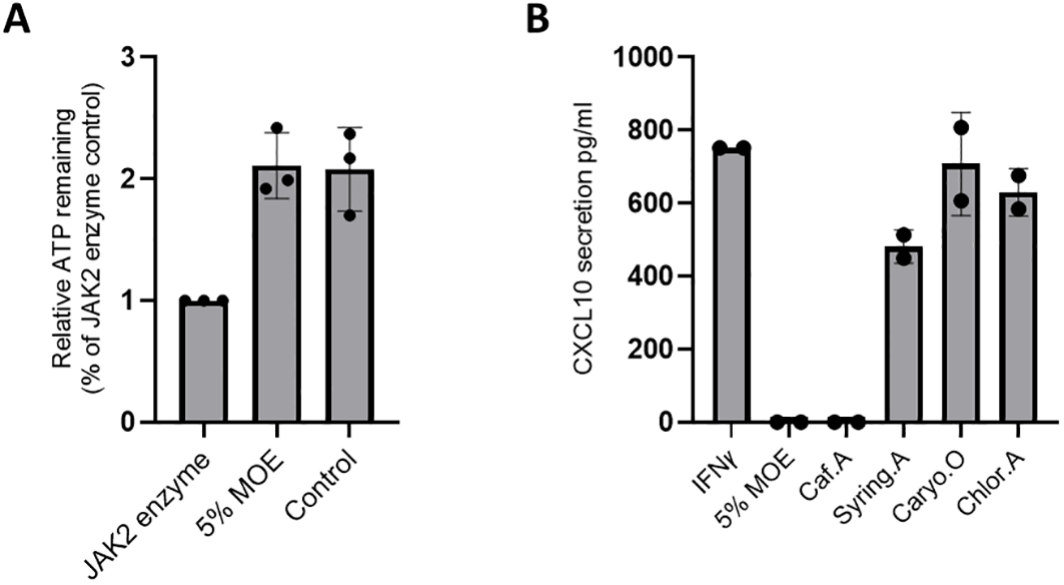
Selected Melissa officinalis constituents partially reproduceMOE-mediated CXCL10 inhibition. **(A)** JAK2 kinase activity was measured using a luminescence-based kinase assay. Luminescence reflects ATP remaining after the kinase reaction. Higher values indicate greater residual ATP and therefore lower JAK2 kinase activity. Data were normalized to the JAK2 enzyme control (100%). Data are presented as mean ± SD (n = 3 independent experiments). No statistical test was performed. **(B)** CXCL10 protein levels in cell culture supernatants measured by ELISA following treatment for six hours with 5% MOE, and 25 µM of caffeic acid, syringic acid, caryophyllene oxide, and chlorogenic acid (n = 2 independent experiments). These data are preliminary and require confirmation with additional replicates. *Caf.A, caffeic acid; Syring.A, syringic acid; Caryo.O, caryophyllene oxide; Chlor.A, chlorogenic acid*.

To investigate whether MOE can affect JAK/STAT signaling upstream of STAT1, a cell-free JAK2 kinase activity assay was performed. MOE produced strong inhibition of JAK2 kinase activity under these assay conditions. Luminescence in this ATP-based kinase assay is directly proportional to the amount of ATP remaining after the reaction and therefore inversely proportional to JAK2 kinase activity. Compared with the JAK2 enzyme control, the 5% MOE treatment exhibited significantly higher normalized luminescence (approximately 2-fold), indicating reduced ATP consumption and inhibition of JAK2 kinase activity, indicating near-complete inhibition in this cell-free assay (**Figure 7A**). As expected, the no-enzyme control also showed high luminescence because ATP was not consumed during the reaction. These findings show that MOE can suppress JAK2 kinase activity *in vitro*, but they do not establish JAK2 as the only or primary intracellular target of the crude extract.

### Candidate Melissa officinalis constituents reproduce MOE-mediated inhibition of JAK/STAT-associated signaling

To evaluate which Melissa officinalis constituents may contribute to the observed modulation of JAK/STAT-associated signaling, CXCL10 secretion was examined following treatment with selected compounds using ELISA (**Figure 7B**). Caffeic acid demonstrated the strongest inhibitory effect, whereas syringic acid showed moderate activity and chlorogenic acid exhibited limited inhibition. Caryophyllene oxide produced variable responses across replicates. These exploratory findings suggest that caffeic acid may contribute to the CXCL10-suppressive effects observed with MOE, but the low number of independent experiments and the complexity of the crude extract require cautious interpretation. To exclude cytotoxic masking of CXCL10 inhibition, HSC2 cells were exposed to 25 µM of the four candidate compounds and viability was confirmed using MTT (**Supplementary Figure 4**).

## Discussion

Oral inflammatory diseases such as OLP involve sustained epithelial-immune communication, in which interferon-associated JAK/STAT signaling can amplify chemokine production and cellular inflammatory responses. The present study therefore focused on whether MOE broadly suppresses inflammatory activation or instead modulates defined signaling outputs in oral epithelial cells. The transcriptomic data support the latter interpretation. In IL-1β/TNF-α-stimulated HSC2 cells, MOE reduced a coordinated interferon-stimulated gene program including MX1/2, IFIT1/3/5, OAS1-3, ISG15, GBP1, STAT1, STAT2, IRF7, CXCL10, and CXCL11. This pattern is consistent with inhibition of a secondary interferon amplification program rather than a global shutdown of cytokine responses. Although IL-1β and TNF-α do not directly engage the IFNγ receptor, TNF can drive IRF1-dependent autocrine interferon signaling and sustained expression of STAT1-dependent chemokines (34). CXCL10 is particularly informative because its transcription depends on cooperation between NF-kB-linked inflammatory inputs and interferon-regulatory mechanisms (35). The selective reduction of CXCL10, together with unchanged CXCL8 expression, therefore identifies MOE as a modulator of interferon-associated epithelial inflammation rather than as a nonspecific anti-inflammatory extract.

The interferon-selective transcriptomic signature was supported by cellular experiments using canonical IFNγ stimulation. IFNγ induced a strong pSTAT1-associated nuclear signal in epithelial cells, whereas MOE reduced both the phosphorylation-associated signal and nuclear localization of STAT1. This effect was functionally reflected by reduced IFNγ-induced CXCL10 and GBP1 expression, linking the imaging readout to downstream interferon-responsive gene transcription. The cell-free JAK2 assay further showed that MOE can inhibit JAK2 kinase activity under defined biochemical conditions, providing a plausible upstream explanation for impaired STAT1 activation. These findings should be interpreted cautiously because the crude extract contains multiple constituents and the cellular target cannot be assigned to JAK2 alone. Nevertheless, the convergence of RNA sequencing, RT-qPCR, ELISA, immunofluorescence, and kinase activity data supports a coherent molecular model in which MOE attenuates the IFNγ-JAK/STAT1-CXCL10 axis. Importantly, the effect was reproduced at the level of pSTAT1 nuclear translocation in primary oral keratinocytes, indicating that the observed response is not restricted to the transformed HSC2 model, although broader validation in non-transformed cells from multiple donors remains necessary.

The phytochemical profile provides a plausible but not definitive basis for the observed cellular activity. MOE contained phenolic acids and terpenoid-related constituents, including caffeic acid, syringic acid, and caryophyllene oxide, which have been linked in previous studies to modulation of JAK/STAT-associated signaling (22–27). In the present experiments, caffeic acid reduced CXCL10 secretion and therefore emerged as a candidate contributor to the activity of the crude extract. However, this compound-level analysis remains exploratory, and the biological activity of MOE cannot be attributed to a single constituent without fractionation, reference-standard confirmation of all relevant metabolites, and systematic testing of individual compounds and combinations. The ROS experiments further refined the mechanism. MOE did not increase intracellular ROS, and NAC did not rescue MOE-mediated suppression of STAT1 nuclear localization or CXCL10/GBP1 expression. Thus, the inhibitory effect on interferon signaling is unlikely to be explained by general redox modulation. Instead, the data point toward a more specific interference with signaling events upstream or at the level of STAT1 activation, involving JAK2, additional JAK family members, IRF-dependent secondary interferon loops, or the STAT1/STAT2 transcriptional complex.

The cellular findings are relevant because epithelial cells are increasingly recognized as active regulators of mucosal immune responses rather than passive targets of inflammation (4). Within this framework, a selective reduction of interferon-associated epithelial outputs may be biologically meaningful even without broad inhibition of NF-kB-linked cytokine responses. CXCL10 was validated at both mRNA and protein levels and represents the strongest downstream readout in this study. This is relevant to OLP biology because CXCL10 has been identified among highly regulated genes in OLP-associated datasets (36), and CXCL9, CXCL10, and CCL19 have been reported to cooperate in T-cell recruitment in lichen planus (37). Increased CXCL10-positive macrophages in OLP tissues further support the importance of this chemokine axis in the inflammatory microenvironment (38). These observations do not establish clinical efficacy of MOE, but they place the epithelial response observed here into a disease-relevant molecular context. They also suggest that future studies should examine epithelial-immune cocultures, T-cell recruitment, and macrophage-associated CXCL10 networks to determine whether the epithelial suppression observed *in vitro* translates into altered immune-cell behavior.

Several limitations should be considered when interpreting the present data. Most experiments were performed in HSC2 oral squamous carcinoma cells, which are useful for mechanistic screening but do not fully recapitulate normal oral epithelial biology; therefore, confirmation in additional primary keratinocyte donors, organotypic epithelial models, and *in vivo* validation are required. The use of epithelial–immune co-culture models would help determine whether the observed epithelial suppression translates into altered immune cell behavior and would better mimic the biological microenvironment of oral lichen planus (OLP). STAT1 inhibition was assessed mainly by immunofluorescence-based detection of pSTAT1 nuclear localization, supported by downstream gene expression and JAK2 activity data, but Western blot analysis of total STAT1 and pSTAT1 would provide additional validation. The JAK2 assay demonstrates kinase inhibition in a cell-free system, yet it does not prove that JAK2 is the only or primary intracellular target of MOE. Future studies should investigate whether MOE similarly modulates other JAK family members, including JAK1, JAK3, and TYK2, to better characterize its mechanism of action. Likewise, HPLC-HRMS identified candidate constituents, but their individual contributions within the crude extract remain speculative. Taken together, the study identifies MOE as a pathway-selective modulator of interferon-associated signaling in oral epithelial cells. The strongest evidence is the convergence of transcriptomic suppression of interferon-stimulated genes, reduced CXCL10 mRNA and protein expression, impaired STAT1 nuclear activation, and ROS-independent inhibition of the IFNγ-JAK/STAT1 response.

The present findings provide mechanistic support for the traditional use of *Melissa officinalis* in oral inflammatory conditions and identify selective modulation of the JAK/STAT pathway as a potential therapeutic mechanism. These results support further development of standardized topical *Melissa officinalis* formulations for oral mucosal diseases. Future studies should evaluate efficacy in more physiologically relevant models and *in vivo* systems, followed by well-designed clinical trials to establish the safety and therapeutic potential of *Melissa officinalis* in patients with oral inflammatory disorders.

## Data Availability

The original RNA-sequencing data generated in this study are publiclyavailable in the European Nucleotide Archive under BioProject accessionPRJEB114350 and study accession ERP194657: https://www.ebi.ac.uk/ena/browser/view/PRJEB114350. Additional data supporting the conclusions of this article are includedin the article and its **Supplementary Material**.
