# Supplementary material for: Melissa officinalis extract selectively suppresses STAT1 signaling in oral epithelial cells
Source: Front Immunol. 2026 Jul 17;17:1892750. doi: 10.3389/fimmu.2026.1892750 (PMC13425913; doi:10.3389/fimmu.2026.1892750)
Supplement: Supplementary file 1 [file DataSheet1.zip › R1_Supplementary Files/R1_Supplementary Figures.docx]

**Supplementary Figures**

*
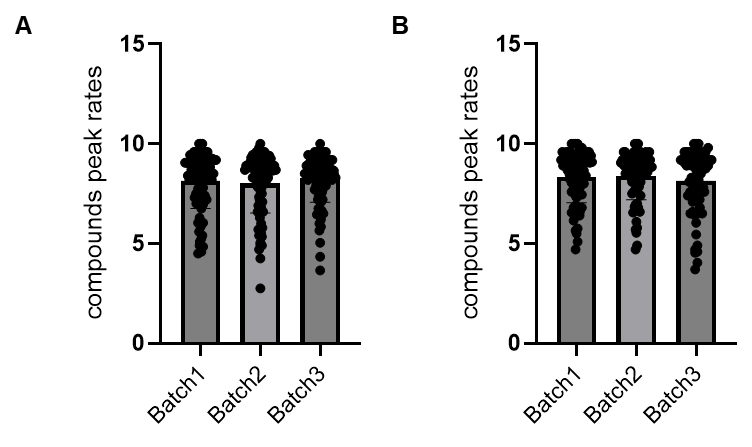
*

*Supplementary Figure 1: Batch to batch consistency assessment of MOE*

Three independently prepared MOE batches were analysed by HPLC-HRMS in A) positive (ESI⁺) and B) negative (ESI⁻) ionization modes. Relative peak areas of identified compounds were compared across batches to assess extraction reproducibility. No significant differences were observed between batches in either ESI⁺ or ESI⁻ modes, indicating consistent chemical profiles across independent preparations. Statistical analysis was performed using one-way ANOVA. Data are presented as mean ± SD.

*
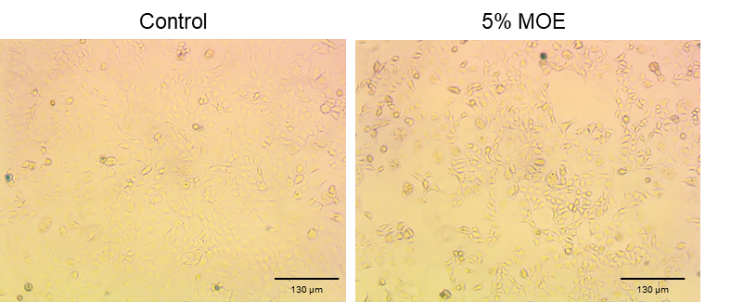
*

*Supplementary Figure 2. MOE does not affect viability of HSC2 cells*

HSC2 cells were exposed to 5% (v/v) MOE for 24 hours. Cell viability was assessed by Trypan blue exclusion assay, where viable cells remained unstained and non-viable cells appeared blue (scale bar = 130 µm). The assay confirmed that 5% (v/v) MOE did not visibly increase cell death under the conditions used for subsequent experiments.

*
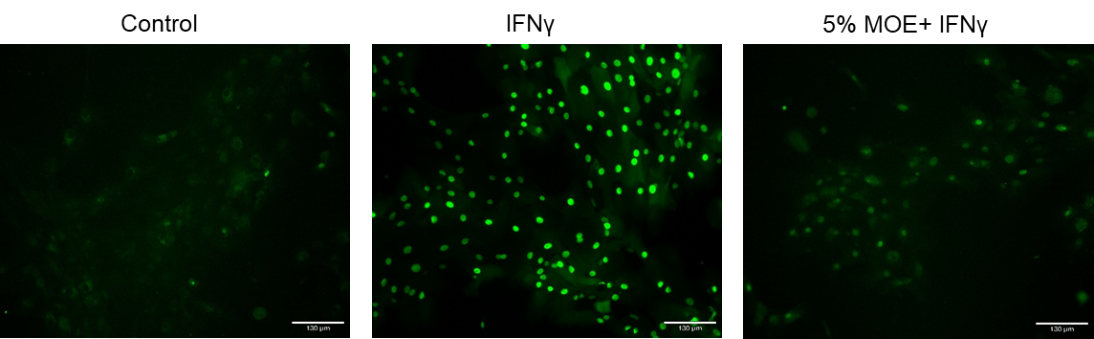
*

*Supplementary Figure 3. Effect of MOE on pSTAT1 nuclear translocation in primary oral keratinocytes.*

Primary oral epithelial cells were stimulated for 1 h with IFNγ, 5% (v/v) MOE, or a combination of both treatments. Following stimulation, cells were fixed and immunostained for phosphorylated STAT1 (pSTAT1) to assess nuclear translocation. Representative images show that MOE reduced IFNγ-induced pSTAT1 nuclear localization in non-transformed oral epithelial cells.

*
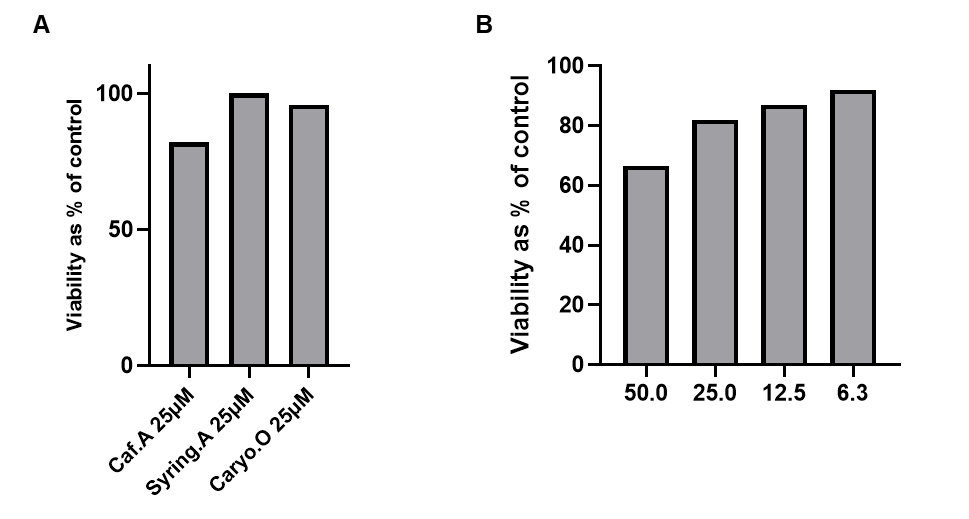
*

*Supplementary Figure 4. Melissa officinalis constituents do not affect HSC2 cell viability*

HSC2 cell viability following exposure to selected Melissa officinalis constituents was assessed by MTT assay and expressed as a percentage relative to the untreated control. HSC2 cells were treated with increasing concentrations of the compounds for 6 h. (A) Cell viability following treatment with 25 µM caffeic acid, syringic acid and caryophyllene oxide. (B) Cell viability following treatment with increasing concentrations of caffeic acid. Data confirm that the tested concentrations did not significantly affect cell viability. *Abbreviations: Caf.A, caffeic acid; Syring.A, syringic acid; Caryo.O, caryophyllene oxide.*


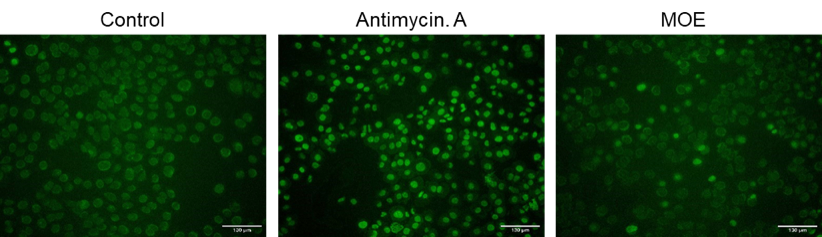


*Supplementary Figure 5. MOE does not induce intracellular ROS*

Intracellular ROS levels measured by CellROX Green fluorescence. HSC2 cells were treated for three hours with 5% (v/v) MOE alone, or antimycin A (positive control, 50 µM), with untreated cells as a negative control. MOE did not elevate ROS levels compared to untreated controls, whereas antimycin A induced a significant increase in ROS.


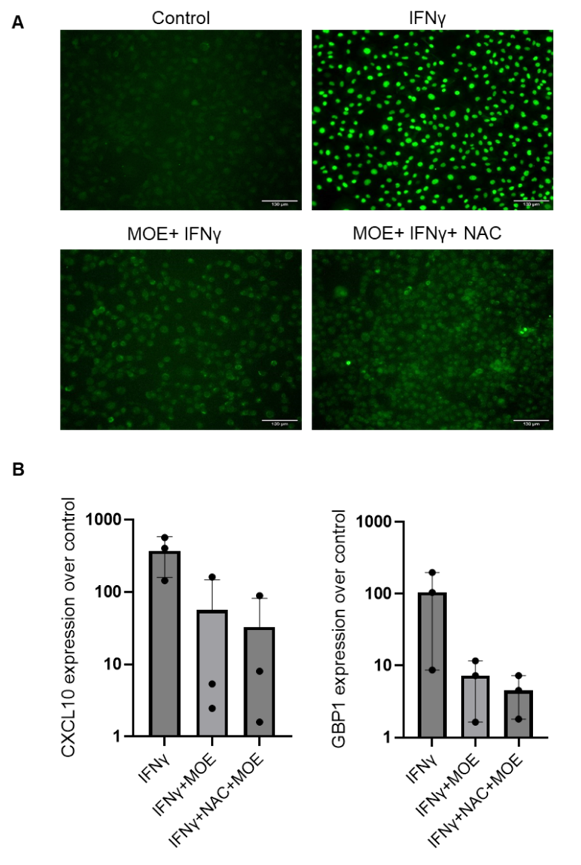


*Supplementary Figure 6. MOE suppresses STAT1 signaling independently of ROS*

(A) Representative immunofluorescence images of STAT1 in HSC2 cells stimulated with IFNγ in the presence or absence of 5% (v/v) MOE and/or NAC (5 mM). IFNγ induced pronounced STAT1 nuclear translocation. MOE treatment markedly reduced STAT1 nuclear localization. Co-treatment with NAC did not restore STAT1 translocation, indicating that the effect of MOE is not mediated by ROS. (B) CXCL10 mRNA expression measured by RT-qPCR in HSC2 cells stimulated with IFNγ in the presence or absence of 5% (v/v) MOE and/or NAC (5 mM). MOE significantly reduced IFNγ-induced CXCL10 expression. NAC did not reverse the suppressive effect of MOE. Data are presented as median ± SD (n = 3 independent experiments).
